# Does portal vein anatomy influence intrahepatic distribution of metastases from colorectal cancer?

**DOI:** 10.2478/raon-2024-0039

**Published:** 2024-09-15

**Authors:** Anaïs Tribolet, Maxime Barat, David Fuks, Mathilde Aissaoui, Philippe Soyer, Ugo Marchese, Martin Gaillard, Alexandra Nassar, Jean Hardwigsen, Stylianos Tzedakis

**Affiliations:** Department of Digestive Surgery and Liver Transplantation, La Timone Hospital, AP-HM, University Aix-Marseille, Marseille, France; Department of Hepatobiliary, Digestive and Endocrine Surgery, Cochin Hospital, AP-HP, Paris, France; Université Paris Cité, Faculté de Médecine, Paris, France; Department of Radiology, Cochin Hospital, Paris, France

**Keywords:** colorectal liver metastases, portal vein anatomy, liver topography, portal variations, portal flow

## Abstract

**Background:**

Other than location of the primary colorectal cancer (CRC), a few factors are known to influence the intrahepatic distribution of colorectal cancer liver metastases (CRLM). We aimed to assess whether the anatomy of the portal vein (PV) could influence the intrahepatic distribution of CRLM.

**Patients and methods:**

Patients with CRLM diagnosed between January 2018 and December 2022 at two tertiary centers were included and imaging was reviewed by two radiologists independently. Intra-operator concordance was assessed according to the intraclass correlation coefficient (ICC). The influence of the diameter, angulation of the PV branches and their variations on the number and distribution of CRLM were compared using Mann-Whitney, Kruskal-Wallis, Pearson's Chi-square and Spearman's correlation tests.

**Results:**

Two hundred patients were included. ICC was high (> 0.90, P < 0.001). Intrahepatic CRLM distribution was right-liver, left-liver unilateral and bilateral in 66 (33%), 24 (12%) and 110 patients (55%), respectively. Median number of CRLM was 3 (1–7). Type 1, 2 and 3 portal vein variations were observed in 156 (78%), 19 (9.5%) and 25 (12%) patients, respectively. CRLM unilateral or bilateral distribution was not influenced by PV anatomical variations (P = 0.13), diameter of the right (P = 0.90) or left (P = 0.50) PV branches, angulation of the right (P = 0.20) or left (P = 0.80) PV branches and was independent from primary tumor localisation (P = 0.60). No correlations were found between CRLM number and diameter (R: 0.093, P = 0.10) or angulation of the PV branches (R: 0.012, P = 0.83).

**Conclusions:**

PV anatomy does not seem to influence the distribution and number of CRLM.

## Introduction

Colorectal cancer (CRC) is the third most common cancer worldwide, with more than two million affected patients in 2020, and the second most common cause of death, with more than one million deaths per year.^1^ The liver is the most common organ of dissemination in patients with CRC. Between 15% and 25% of patients with CRC are diagnosed with synchronous liver metastasis at the time of diagnosis; while up to 25% patients with non-metastatic CRC will develop liver metastases within five years following initial diagnosis.^2,3^ In association with chemotherapy, surgery remains the only curative option for patients with colorectal liver metastases (CRLM).^4^ Considerations when assessing resectability of CRLM usually take into account technical aspects such as tumour relationship to vascular inflow, outflow, and biliary drainage but also liver disease burden (i.e. size and number).^5^

The number and size of CRLMs are well-known prognostic markers of disease^5,6^, however, little is known about the factors influencing CRLM intrahepatic distribution. Primary colorectal cancer localization has already been described as a factor determining CRLM intrahepatic distribution, although evidence is conflicting. Several authors have hypothesized that the portal vein (PV) ‘streamline flow’, resulting in cells moving in different layers, may influence CRLM intrahepatic distribution.^7,8,9^ Indeed, since the PV is formed by the confluence of the superior mesenteric and splenic vein, it has been theorized that venous blood flow from mesenteric veins mix incompletely in the PV resulting in a disproportionate lobar distribution within the liver due to superior mesenteric venous drainage preferentially directed towards the right liver.^10^

On the other hand, it has already been shown that right portal vein (RPV) diameter is larger than left portal vein (LPV) diameter (principally related to the higher right liver volume)^11,12^ and that PV flow volume tends to change in proportion to changes in PV cross-sectional area^13^, thus creating disproportionate flow volume and metastatic potential between the two hemi-livers. Finally, little is known on the influence that PV variations, found in up to 20–35% of individuals, might have in flow volume changes and CRLM distribution.^14,15^ The development of CRLM is a multifactorial process and knowledge of potential PV anatomy influence on the CRLM distribution could enable to anticipate and tailor patient management and surveillance. Indeed, a high metastatic burden of the right or left liver related with PV variations could influence the choice of an anatomical liver resection (due to the higher risk of missing lesions) or parenchyma-sparing liver surgery in case of multiple CRLM. Since no study has assessed the influence of PV diameter and consequently flow volume on CRLM intrahepatic distribution so far, the aim of the present study was to evaluate the influence of PV parameters and anatomical variations on intrahepatic distribution of CRLM.

## Patients and methods

### Study population

Between January 2018 and December 2022, data of all consecutive patients undergoing curative liver resection for CRLMs were retrieved from a prospectively collected database at two tertiary hepatobiliary centres. Histologic confirmation of CRLM was obtained by examination of resected specimens. Patients with previous hepatectomy and those for whom computed tomography (CT) and magnetic resonance imaging (MRI) were not available or uninterpretable were excluded. Previous primary CRC resection was not an exclusion criterion. Patients with histologically proven cirrhosis and those presenting with severe liver dysmorphy or segmental/lobar atrophy were also excluded.^16^ This study was approved by the institutional review board (number: AAA-2023-09046).

### Patient and tumour characteristics

The collected data included baseline patients' demographic data, primary tumour location, synchronous or metachronous diagnosis of CRLM, resected primary CRC, tumour node metastasis (TNM) classification tumour stage and RAS, BRAF tumour mutational status as well as microsatellite instability (MSI) or stability (MSS) status. Patients with primary CRC located between the cecum and transverse colon were included in the right-sided colon group while patients with CRC located between the splenic flexure and the recto-sigmoid junction were included in the left-sided colon group. Rectal cancer was divided into high rectum group and a low and medium rectum group, related to different venous drainage axes.^17^

### Computed tomography imaging and analyses

For this retrospective study, each set of CT and MRI images of individual patients of the two centres were reviewed, in a random manner, following anonymization using a picture archiving and communication system workstation (DirectView, v. 11.3, Carestream Health, Rochester, NY) by two radiologists (with 12 and 10 years of experience in abdominal imaging, respectively).

All abdominal CT and MRI examinations were performed before induction of chemotherapy to decrease missing-metastasis biases using a multidetector CT (64-detector) scanners from different manufacturers. CT acquisitions covered the entire abdomen and pelvis. To examine the anatomy of the PV, all examinations included at least an acquisition during the portal-venous phase performed after a delay of 70 to 80 s after intravenous administration of iodinated contrast material. Portal-venous phase was defined when all portal branches and hepatic veins were fully enhanced. The number of CRLM was assessed using MRI examinations including T2-weighted sequences, diffusion weighted sequences, T1 DIXON weighted sequences and T1 with fat saturation with and without gadolinium-chelate enhanced images. MR images were acquired on a 1.5T Siemens Avanto (centre 1 and 2) and a 3T Siemens Skyra (centre 1) scanner. CT exams were acquired using three dimensional acquisitions, thickness of 0.6mm, automatic z-axis-modulation and optimized noise. Characteristics of the different MRI protocols in the two centres are summarized in Supplementary Table 1.

### Portal vein anatomy and CRLM intrahepatic distribution

**Figure 1.**
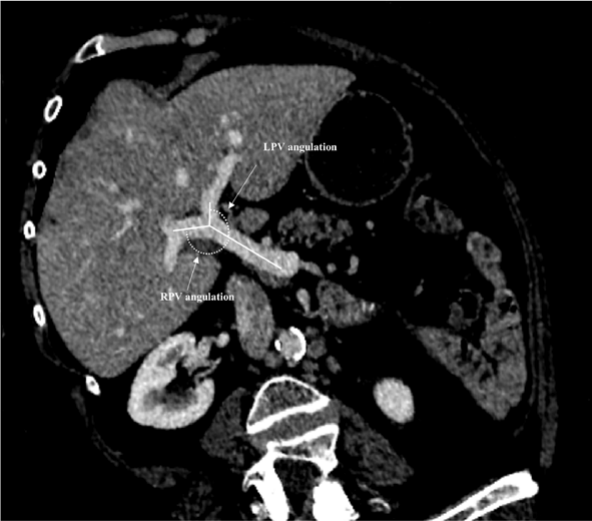
Measurements of right portal vein and left portal vein angulation. RPV = right portal vein, LPV = left portal vein

Common anatomic variations of the PV were identified and recorded as previously described.^5,18^ Normal PV anatomy included division of the PV into right and left branches immediately before reaching the liver, with further division of the right portal branch into anterior and posterior sectorial branches (Type 1). Type 2 PV variation included PV trifurcation with left portal branch and both right sectorial portal branches sharing the same origin. Type 3 variation included right anterior sectorial branch arising from the left PV. The following imaging PV parameters were finally recorded: (1) The primitive branch of the PV presenting the largest cross-sectional area was also recorded and defined as the predominant PV branch (right or left). (2) PV diameter of the main PV, the primitive RPV and LPV measured just before and after main PV bifurcation using previously published methods.^19^ (3) RPV and LPV angulations were measured as follows: after three-dimensional-reconstruction in the plane of the PV, the angle between the last segment of the PV and the initial segment of RPV and LPV lumens were calculated using previously published methods (Figure 1).^20^ For type 2 portal anatomy variations, the diameter of the right and left anterior sectorial branches were summed up and angulations averaged for the right liver. For patients with a type 3 PV variation CRLM localized in the anterior sector (segments 5 and 8) were attributed to the left liver, given the fact that right anterior segmental branch originated from the LPV.

Intrahepatic distribution of CRLM was recorded as follows: the number of CRLM as well as their segmental location were recorded according to the Couinaud segment classification.^21^ The left liver was composed of segments 2, 3, 4 while the right liver included segments 5, 6, 7 and 8. Lesions located in segment 1 were considered independently given the specificity of portal vein vascularization.^22^

### Statistical analysis

The distribution of quantitative variables was assessed using the Shapiro-Wilk test. Quantitative variables were reported as means ± standard deviations (SD) or medians with 25–75 interquartile range (IQR) depending on their distribution^23^ and were compared using the Mann-Whitney, Kruskal-Wallis or Student t-test as appropriate. Categorical variables were expressed as raw numbers, proportions and percentages and were compared using Pearson's Chi-square or Fisher's exact test as appropriate. Intra-class correlation coefficient (ICC) based on a two-way random effects model was used to determine the reliability of the measurements between the two radiologists.^24^ ICC between 0.00 and 0.20; 0.21 and 0.40; 0.41 and 0.60; 0.61 and 0.80; and 0.81 and 1.00, indicated slight, fair, moderate, substantial, and almost perfect agreement. Correlations between portal vein diameter, angulation and number of CRLM in the relevant liver segment were evaluated using Spearman correlation tests. Sensitivity analysis involved subgroup comparison of patients presenting only unilateral (right and left) CRLM and patients presenting a single CRLM to account for early stage of disease. All statistical tests were two-tailed, with P < 0.05 considered to indicate statistically significant differences. All analyses were performed using RStudio statistical software (Version 1.4.1103 © 2009–2021 RStudio, Inc).

## Results

### Study population and baseline characteristics

**Figure 2.**
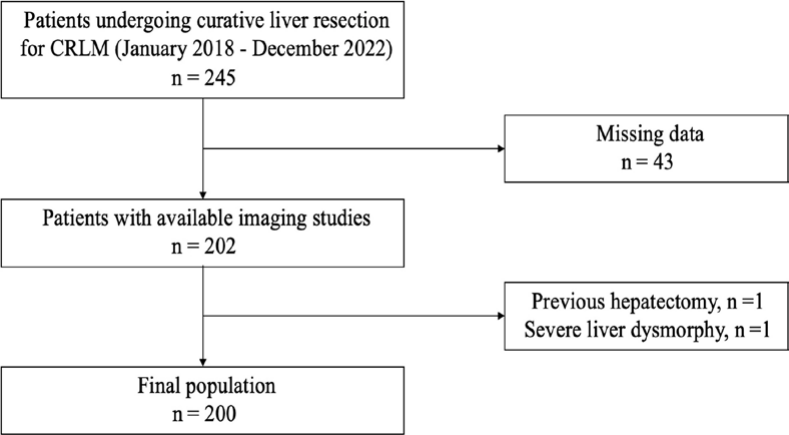
Patient flowchart. CRLM = colorectal liver metastasis

During the study period, 245 patients were diagnosed with CRLM. Among these patients, 45 (18%) patients were excluded due to missing data (n = 43), previous hepatectomy (n = 1) and presence of liver dysmorphia related to cirrhosis (n = 1). The final population included 200 patients (Figure 2). Median age was 64 years (Q1, Q3: 57, 71) and 41% were females. CRLM were predominant in the right liver with 807 (65%) lesions compared to 436 (35%) lesions in the left liver (P < 0.01). The majority of patients (70%) presented with synchronous CRLM and were more often bilateral than unilateral (55% vs. 45%; P = 0.01). Among patients with unilateral lesions 24 (12%) were localized only in the left liver and 66 (33%) only in the right liver. Primary tumour location was left-sided in 112 (57%) patients, right-sided in 34 (17%) patients and rectal in 52 (26%) patients. The majority of patients (82%) presented a T3–T4 primary tumour stage. Patient characteristics were similar between unilateral right, left and bilateral CRLM and are detailed in Table 1.

**Table 1.** Bilateral, right and left unilateral distribution of colorectal liver metastases and vascular anatomy

**Characteristics**	**Overall, N = 200^a^**	**Bilateral N =110 (55%)^a^**	**Right unilateral N = 66 (33%)^a^**	**Left unilateral N = 24 (12%)^a^**	**P-value^b^**
**Patient**					
**Age (years)**	64 (57–71)	63 (53–70)	65 (58–74)	67 (63–71)	**0.02**
**Gender: female**	81 (41)	47 (43)	29 (44)	5 (21)	0.11
**BMI (kg/m2)**	24 (21.0–27.0)	24 (21.0–27.0)	24 (22.0–26.0)	24 (23.0–27.3)	0.70
**Primitive tumor location**					0.60
Right colon	34 (17)	16 (15)	12 (18)	6 (25)	
Left colon	112 (57)	60 (56)	38 (58)	14 (58)	
Rectum	52 (26)	32 (30)	16 (24)	4 (17)	
**T stage**					0.60
0–2	31 (18)	15 (17)	10 (17)	6 (26)	
3–4	139 (82)	74 (83)	48 (83)	17 (74)	
**N stage**					0.80
0	66 (40)	33 (38)	23 (43)	10 (43)	
1–2	98 (60)	55 (63)	30 (57)	13 (57)	
**KRAS mutation**	60 (30)	33 (30)	20 (30)	7 (29)	> 0.90
**BRAF mutation**	6 (3)	4 (3.6)	2 (3)	0 (0)	> 0.90
**MSI**	4 (2)	2 (1.8)	1 (1.5)	1 (4.2)	0.60
**Number of CRLM**	3 (1–7)	6 (4–11)	1 (1–3)	1 (1–1)	**< 0.01**
**Synchronous CRLM**	140 (70)	87 (79)	38 (58)	15 (63)	**0.01**
**Vascular**					
**PV variations**					0.13
Type 1	156 (78)	88 (80)	51 (77)	17 (71)	
Type 2	19 (9,5)	13 (12)	3 (4.5)	3 (13)	
Type 3	25 (13)	9 (8.2)	12 (18)	4 (17)	
**PV diameter**					
Main trunk	13.5 (12.4–15)	13.3 (12.4–15)	13.7 (12.50–15)	13.8 (12.15–14.20	>
RPV	11.3 (10.0–12.9)	11.4 (10.0–13.0)	11.3 (10.0–12.6)	11.3 (10.3–12.1)	0.90
LPV	10.7 (9.6–12.0)	10.7 (9.6–12.0)	10.5 (9.0–11.9)	10.9 (10.1–12.0)	0.50
**Ratio diameter RPV/LPV**	1.1 (0.9–1.2)	1.1 (0.9–1.2)	1.1 (1.0–1.3)	1.1 (0.9–1.2)	0.40
**PV branches angulation**					
RPV	153 (142–163)	151 (140–162)	155 (145–168)	158 (147–164)	0.20
LPV	97 (80–115)	98 (77–113)	97 (81–118)	98 (84–116)	0.80
**Predominant PV branch^c^**				0.50	
RPV	123 (67)	67 (67)	43(70)	13 (57)	
LPV	61 (33)	33 (33)	18(30)	10 (43)	
**Arterial variations**					
Right hepatic artery	22 (11)	13 (12)	8 (12)	1 (4.2)	0.70
Left hepatic artery	24 (12)	12 (11)	8 (12)	4 (17)	0.70

a = n (%), median (interquartile range, IQR);

b = Fisher's exact test, Kruskal-Wallis;

c = primitive branch of the portal vein presenting the largest cross-sectional area

BMI = body mass index; CRLM = indicates colorectal cancer liver metastasis; LPV = left portal vein; MSI = microsatellite instability; PV = portal vein; RPV = right portal vein

Type 1 portal vein anatomy was most frequently observed with 156 (78%) patients while 19 (9.5%) patients had a type 2 and 25 (12%) patients a type 3 variation. Arterial variations consisted in the presence of an accessory left hepatic artery in 24 (12%) patients and an accessory right hepatic artery in 22 (11%) patients. The median diameter of the main PV was 13.5 mm (Q1, Q3: 12.4, 15), and RPV 11.3 mm (Q1, Q3: 10, 12.9) was significantly larger than the LPV 10.7 mm (Q1, Q3: 9.6, 12) (P = 0.002). Overall, 123 (61.5%) patients presented a predominant (branch with the largest cross-sectional area) RPV and 61 patients (30.5%) a predominant LPV, while 16 (8%) patients presented an identical diameter. The median angulation of the RPV and LPV was 153° (Q1, Q3: 142, 163°) and 97° (Q1, Q3: 80, 115°), respectively (P < 0.001). Liver vascular anatomy is detailed in Table 1.

### Portal vein anatomy and intrahepatic distribution of colorectal cancer liver metastases

ICC between radiologists was excellent in the evaluation of the number and intrahepatic distribution of CRLM (0.99; 95% CI: 0.99–1.00; P < 0.001) and PV angulation measurements (0.97; 95% CI: 0.93–0.99; P < 0.001) and was very good for PV diameter and variations measurements (0.86; 95% CI: 0.73–0.99; P < 0.001).

The median diameter of the main PV in bilateral, unilateral right and left liver CRLM was 13.3 mm (Q1, Q3: 12.4, 15), 13.7 mm (Q1, Q3: 12.5, 15) and 13.8 mm (Q1, Q3: 12.1, 14.2), respectively and no significant differences were found (P > 0.90). Accordingly, the median RPV diameter was 11.4 mm (Q1, Q3: 10, 13), 11.3 mm (Q1, Q3: 10, 12.6) and 11.3 mm (Q1, Q3: 10.3, 12.1) (P = 0.90) and median LPV diameter was 10.7 mm (Q1, Q3: 9.6, 12), 10.5 mm (9, 11.9) and 10.9 mm (Q1, Q3: 10.1, 12) (P = 0.50). Concerning PV branch angulations, the median angulation of the RPV was 151° (Q1, Q3: 140, 162) for the bilateral distribution of CRLM, 155° (Q1, Q3: 145 – 168) for the right unilateral, 158° (Q1, Q3: 147, 164) for the left unilateral (P = 0.20) and the median angle of the LPV was 98° (Q1, Q3: 77, 113) for the bilateral distribution of CRLM, 97° (Q1, Q3: 81, 118) for right unilateral and 98° (Q1, Q3: 84, 116) for left unilateral (P = 0.80). Finally, unilateral - bilateral CRLM intrahepatic distribution was also independent of PV variations. Type 1 anatomy of the PV was present in 88 patients (80%) for bilateral, in 51 patients (77%) for unilateral right and in 17 patients (71%) for unilateral left distribution (P = 0.13). Characteristics of the PV anatomy according to bilateral, unilateral right and left CRLM intrahepatic distribution are detailed in Table 1.

In subgroup analysis, considering only patients with unilateral CRLM or patients with a predominant RPV or LPV no significant associations between the intrahepatic distribution of CRLM and PV anatomy were found. Subgroup left unilateral versus right unilateral analysis and RPV versus LPV predominance are detailed in Table 2 and 3, respectively.

**Table 2.** Unilateral right and left distribution of colorectal liver metastases and portal anatomy

**Characteristics,**	**Right unilateral, N = 66 (73%)^a^**	**Left unilateral, N = 24 (27%)^a^**	**p-value^b^**
**Number of CRLM**	1 (1–3)	1 (1–1)	**0.03**
**PV variations**			
Type 1	51 (77)	17 (71)	
Type 2	3 (4.5)	3 (13)	
Type 3	12 (18)	4 (17)	
**PV diameter**			
Main trunk	13.7 (12.5–15.0)	13.8 (12.2–14.2)	0.70
RPV	11.3 (10.0–12.6)	11.3 (10.3–12.1)	0.60
LPV	10.5 (9.0–11.9)	10.9 (10.1–12)	0.20
**Ratio diameter RPV/LPV**	1.1 (1.0–1.3)	1.1 (0.9–1.2)	0.20
**PV branches angulation**			
RPV	155 (145–168)	158 (147–164)	> 0.90
LPV	97 (81–118)	98 (84–116)	0.90
**Predominant PV^c^**			0.30
RPV	43 (70)	13 (57)	
LPV	18 (30)	10 (43)	

a = n (%), median (interquartile range, IQR);

b = Fisher's exact test, Kruskal-Wallis;

c = primitive branch of the portal vein presenting the largest cross-sectional area

CRLM = colorectal liver metastasis; LPV = left portal vein; PV = portal vein; RPV = right portal vein

**Table 3.** Predominance of right portal vein (RPV) or left portal vein (LPV) and intrahepatic distribution of colorectal liver metastasis (CRLM)

**Characteristics**	**Predominant RPV N = 123 (67%)^a,b,c^**	**Predominant LPV N = 61 (33%)^a,b,c^**	**p-value^d^**
**Distribution of CRLM**			
Bilateral	67 (54)	33 (54)	
Right unilateral	43 (35)	18 (30)	
Left unilateral	13 (11)	10 (16)	
**Number of segments involved (right liver)**	2 (1–3)	2 (1–3)	0.60
**Number of segments involved (left liver)**	1 (0–2)	1 (0–2)	0.70
**Total number of CRLM**	3 (1–8)	4 (1–5)	0.70
**Number of CRLM in the right liver**	2 (1–5)	2 (1–4)	0.40
**Number of CRLM in the left liver**	1 (0–3)	1 (0–2)	0.60
**Ratio number of CRLM right/left liver**	1 (1.0–3.0)	1 (1.0–2.0)	0.70
**Ratio number of CRLM per segment (right liver)**	1 (1.0–2.0)	1 (1.0–2.0)	0.60
**Ratio number of CRLM per segment (left liver)**	1 (1.0–1.8)	1 (1.0–2.0)	> 0.90

a = n (%), median (interquartile range, IQR);

b = primitive branch of the portal vein presenting the largest cross-sectional area;

c = Sixteen patients presented an identical RPV and LPV diameter and were excluded;

d = Fisher's exact test, Wilcoxon rank sum test

Finally, among 56 patients with a single CRLM, 37 patients (66%) presented a single metastasis in the right liver, and 19 patients (34%) presented a single metastasis in the left liver. Right and left intrahepatic distribution was independent of the diameter of the PV (P > 0.90), the RPV (P = 0.60) and LPV (P = 0.50), as well as the RPV (P = 0.50) and LPV (P = 0.50) angulation and PV anatomy variations (P = 0.80).

### Portal vein anatomy variations and number of CRLM

**Figure 3.**
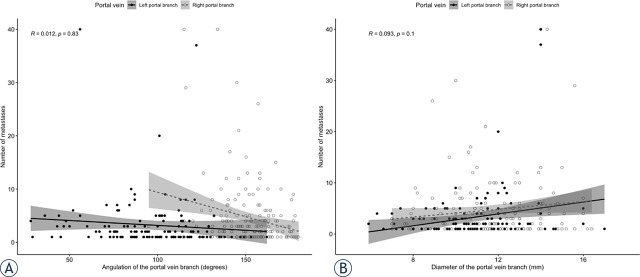
Graphs show correlation between portal vein branches' diameter **(A)**, angulation **(B)** and the number of colorectal liver metastases (CRLM). R = Spearman's correlation coefficient

Overall, the median number of CRLM was 3 (Q1, Q3: 1, 7). The median number of CRLM was significantly greater both in the bilateral CRLM group (6; Q1, Q3: 4, 11) by comparison with those in the unilateral group (1; Q1, Q3: 1, 2) (P < 0.01) and the right unilateral group (1; Q1, Q3: 1, 3) when compared to the left unilateral (1; IQR: 1, 1) (P = 0.03). There was no significant correlation between the number of CRLM in the corresponding hemi-liver and the diameter (R: 0.093; 95% CI: 0.08–0.12; P = 0.10) or the angulation of the PV branches (R: 0.012; 95% CI: 0.009–0.02; P = 0.83). Relation between diameter and PV branches angulation and number of CRLM are presented in Figure 3A and 3B, respectively.

The number of CRLM in the right liver (P = 0.40), in the left liver (P = 0.60) and the number of affected segments in the right (P = 0.60) and in the left liver (P = 0.70) did not significantly differ according to the side of the predominant portal branch.

### Primitive CRC and CRLM intrahepatic distribution

The intrahepatic distribution of CRLM in right-sided CRC was bilateral in 16 (49%) patients, left unilateral for 6 (18%) patients, right unilateral in 11 (33%) patients, whereas for left-sided CRC, CRLM were bilateral in 79 (56%) patients, right unilateral in 47 (33%) patients, left unilateral in 16 (11%) patients (P = 0.60). No differences in median number of total CRLM were found between right-sided (2; Q1, Q3: 1, 6) and left-sided (3; Q1, Q3: 1, 8) CRC was (P = 0.20). Finally, the ratio of right to left liver CRLM was 1 (Q1, Q3: 1.0, 1.3) for right-sided and 1.1 (Q1, Q3: 1.0, 3.0) for left-sided CRC (P = 0.20) (Table 4).

**Table 4.** Location of primary colorectal cancer and intrahepatic distribution of colorectal cancer liver metastases

**–**	**Right colon N = 33 (19%)^a^**	**Left colon N = 142 (81%)^a^**	**p-value^b^**
**Distribution of CRLM**			
Bilateral	16 (49)	79 (56)	
Right unilateral	11 (33)	47 (33)	
Left unilateral	6 (18)	16 (11)	
**Number of CRLM**	2 (1–6)	3 (1–8)	0.20
**Number of CRLM in the right liver**	1 (1–3)	2 (1–5)	0.12
**Number of CRLM in the left liver**	1 (0–2)	1 (0–3)	0.70
**Ratio number of CRLM right/left liver**	1 (1–1)	1 (1–3)	0.20

a = n (%), median (interquartile range, IQR);

b = Fisher's exact test, Wilcoxon rank sum test CRC = colorectal cancer;

CRLM = indicates colorectal liver metastasis

## Discussion

Few factors are known to influence the intrahepatic distribution of CRLM. Primary CRC localization has already been described as a factor determining intrahepatic CRLM distribution, although evidence is conflicting.^8,10,25,26,27^ The aim of this study was to assess whether the anatomy of the portal vein (PV), through variations in blood flow, as well as its anatomical variations could have an influence on the topography of CRLM. No significant difference in the distribution of CRLM in relation with the PV anatomy were identified. Indeed, neither the presence of an anatomical variation of the PV, nor a variation in diameter or angulation of the PV and its branches appeared to have any impact on the unilateral – bilateral distribution or in the number of CRLM. Additionally, in this study we did not find any correlation between the primary tumour location and the intrahepatic distribution of CRLM.

Spread of CRC to the liver is mainly hematogenous through the portal circulation.^28^ The PV is formed by the confluence of the superior mesenteric and splenic vein and its flow volume tends to change in proportion to changes in PV cross-sectional area thus creating disproportionate flow volume and metastatic potential between the two hemi-livers.^13^ Indeed, flow velocity depends on pressure and flow resistance and according to Poiseuille's law, flow resistance depends on the geometry of the tube with the length and radius of the tube. The diameter of the vessel is therefore an important factor, among other parameters, modifying blood flow.^29^ It is known that the diameter of the RPV is larger and the angle more open than the diameter and angle of the LPV.^12^ Diameter has been hypothesized to be linked to a higher blood flow and hemi-liver volume.^12^ Consequently, we hypothesized that since a larger diameter or a more open angle of a portal branch, could lead to an increase in blood flow, it could potentially increase the metastatic potential in the segments vascularized by these branches. Indeed, in our study, patients' weight, height and gender, known parameters influencing the diameter of PV and its branches^19^ were not different and although the total number of CRLM was greater in the right than in the left liver, individual diameter and angulation variations of the PV branches were very small and no direct relation with the number and intrahepatic distribution of CRLM was found. Moreover, anatomical variations of the PV in our population were similar to current literature reports^14,15^ and were also independent of the CRLM intrahepatic distribution.

The results of our study are in line with other studies showing no influence of the primary CRC location on the distribution of CRLM. Several studies have shown an unequal intrahepatic distribution of CRLM depending on the location of the primary CRC and in based on the hypothesis was that CRLM would be distributed differently in the liver due to ‘streamline flow’ in the PV, linked to the different venous drainage of the right and left colon.^7^ Results are not however unequivocal, with some studies reporting an equivalent distribution between right and left liver depending on the location of the primary site^26,27^ and others reporting a preferential distribution of right-sided CRC metastases in the right liver.^8,10,25^ Other intrinsic tumour characteristics have also been described as potential factors influencing CRLM anatomical distribution. A recent study showed that CRLM distribution may differ between different primary tumours as is the case of breast cancer.^30^ In their study, the authors showed that breast cancer most commonly affects the left liver lobe when compared with CRLM and part of the reason seems to be related to its diverse growth cell rate and metastatic potential, tumour size, and histological grade, the knowledge of which can have significant therapeutic implications. Concerning CRLM, although no intrinsic biologic tumour characteristic responsible for the difference in liver metastasis distribution has been clearly identified, gene mutations are known as both prognostic factors for survival and segmental location of the primary CRC^6^ and further studies are needed to extrapolate the role of molecular patterns in CRLM anatomical distribution.

To our knowledge, this is the first study to investigate the effect of different parameters of the portal anatomy over the topography of CRLM. Robustness of our results are supported by independent image analysis by two radiologists with almost perfect inter-operator concordance. Moreover, our sensitivity analyses performed in the subgroup of patients presenting only unilateral and only single CRLM (to compare patients with similar metastatic liver tumour burden) further strengthen our findings.^5^ Nevertheless, this study presents some limitations. Patients are not diagnosed at the same time in the course of their disease, and therefore it is unknown if portal blood flow could be related with the intrahepatic distribution of CRLM at different timepoints of disease. Moreover, patients with synchronous and metachronous liver CRLM were included and already known differences in the oncologic behaviour of synchronous CRLM may have influenced our results. Indeed, patients with synchronous CRLM are known to have a higher number of lesions, a billboard distribution at diagnosis and a worse prognosis.^31^ Furthermore, the underlying liver parenchyma characteristics (cirrhosis, nodular regenerative hyperplasia and ultimately chemotherapy-induced sinusoidal obstruction syndrome) non-available in this study may have further influenced liver venous flow (notably portal vein diameter and portal venous flow increase as already described^32,33^) and thus CRLM distribution regardless of the PV anatomy, however, this bias is likely to be limited due to exclusion of all patients with histologically proven liver cirrhosis.

Although negative, this study could have had an impact in tailoring CRLM surgical management if a potential influence of PV anatomy on CRLM intrahepatic distribution had been confirmed. For example, a hypothetical correlation of right-sided CRLM liver distribution and predominant RPV or type 3 PV anatomy in patients with multiple lesions could have influenced the surgeon's choice to perform an anatomical liver resection (i.e. right-hepatectomy) rather than a parenchyma-sparing liver surgery (if lesions were accessible to both treatments) to lower the high risk of missing lesions in the remaining right liver. Nevertheless, since no significant relationship has been demonstrated, parenchymal sparing liver strategies should remain the first objective whenever possible^34^ enabling repeated liver resections, when necessary, since surgery remains the best curative treatment in CRLM.

In conclusion, the present study did not find any significant impact of the PV anatomy, nor primary CRC tumour location, on the distribution and number of CRLM. Future studies could focus on liver parenchymal and Doppler PV flow characteristics, not studied here, to further investigate potential predictive factors.

## Supplementary Material

Supplementary Material Details
