# Supplementary material for: Does portal vein anatomy influence intrahepatic distribution of metastases from colorectal cancer?
Source: Radiol Oncol. 2024 Sep 15;58(3):376–85. doi: 10.2478/raon-2024-0039 (PMC11406940; doi:10.2478/raon-2024-0039)
Supplement: Supplementary file 1 — Supplementary Material Details [file raon-2024-0039-sm.pdf]

sciendo
Radiology and Oncology | Ljubljana | Slovenia | www.radioloncol.com
RADIOLOGY AND ONCOLOGY

Does portal vein anatomy influence intrahepatic distribution of metastases from colorectal cancer?

Anaïs Tribolet, Maxime Barat, David Fuks, Mathilde Aissaoui, Philippe Soyer, Ugo Marchese, Martin Gaillard, Alexandra Nassar, Jean Hardwigsen, Stylianos Tzedakis

doi: 10.2478/raon-2024-0039

SUPPLEMENTARY TABLE 1. Magnetic resonance imaging sequences and parameters in the two centers

Sequence		Diffusion-weighted	VIBE DIXON	FS T2 BLADE	Dynamic multiphase contrast-enhanced
Plane		Transverse	Transverse	Transverse	Transverse
MR acquisition type		2D	3D	2D	3D
Slice thickness (mm)	Centre 1 1.5T<br>Centre 1 3T<br>Centre 2 1.5T	5<br>5<br>8	3<br>2<br>3	5<br>5<br>5.5	2.2<br>2<br>3
Gap (mm)	Centre 1 1.5T<br>Centre 1 3T<br>Centre 2 1.5T	0.5<br>0.5<br>1.6	-<br>-<br>-	1<br>1<br>1.1	-<br>-<br>-
Repetition time (ms)	Centre 1 1.5T<br>Centre 1 3T<br>Centre 2 1.5T	6000<br>3300<br>1200	6.83<br>3.92<br>6.88	2540<br>3234<br>1200	4<br>3.92<br>4.62
Echo time (ms)	Centre 1 1.5T<br>Centre 1 3T<br>Centre 2 1.5T	56<br>46<br>143	2.4<br>1.23<br>2.39	121<br>105<br>143	1.5<br>1.23<br>2.28
b-value (s/mm^2)		50, 400, 800	-	-	-
Flip angle (°)	Centre 1 1.5T<br>Centre 1 3T<br>Centre 2 1.5T	90<br>90<br>160	10<br>9<br>10	142<br>90<br>160	14<br>9<br>10
Pixel bandwidth (Hz)	Centre 1 1.5T<br>Centre 1 3T<br>Centre 2 1.5T	2440<br>2322<br>475	475<br>930<br>600	500<br>744<br>475	345<br>930<br>400
Acquisition matrix	Centre 1 1.5T<br>Centre 1 3T<br>Centre 2 1.5T	128 × 104/ 140x112<br>256 x 198	320 × 182<br>256 x 146<br>320 x 175	256 × 256<br>320 x 88<br>256 x 198	384 × 234<br>256 x 146<br>320 x 195
Respiratory control		Respiratory-triggered	Breath-hold	Respiratory-triggered	Respiratory-triggered

2D = two-dimensional, 3D = three-dimensional, FS = fat saturated; VIBE = volumetric interpolated breath hold examination

All MR scans are Siemens Healthineers. BLADE is the name of periodically rotated overlapping parallel lines with enhanced reconstruction (PROPELLER) sequence of Siemens Healthcare.
